# Polymorphisms in Glutathione S-Transferase M1, T1, and P1 in Patients with Chronic Periodontitis: A Pilot Study

**DOI:** 10.1155/2014/135368

**Published:** 2014-11-25

**Authors:** Victor Raul Camargo Ortega, Leliette Deyanira Bravo López, Angel Visoso Salgado, Fernando Mejia Sanchez, Julieta Castillo Cadena

**Affiliations:** ^1^Center of Research and Advanced Studies in Dentistry (CIEAO), Autonomous University of the State of Mexico, 50130 Toluca, MEX, Mexico; ^2^Medical Research Center (CICMED), Autonomous University of the State of Mexico, 50130 Toluca, MEX, Mexico

## Abstract

*Background.* Although the direct cause of chronic periodontitis is bacterial infection, the progression of this disease depends on genetic and environmental factors, and smoking is a known risk factor in the development and severity of the disease. An individual's susceptibility may be influenced by polymorphisms in the glutathione S-transferase genes. These genes encode enzymes that metabolize xenobiotic compounds. The aim of this study was to determine the frequency of GSTM1, GSTT1, and GSTP1 polymorphisms in Mexicans with chronic periodontitis. *Methods.* 60 Mexicans with chronic periodontitis (30 smokers and 30 nonsmokers) were studied. A peripheral blood sample was taken for subsequent DNA extraction. The genetic material was PCR-amplified followed by restriction fragment length polymorphism with the aim of identifying GST polymorphisms. *Results.* Polymorphisms in the GSTT1 and GSTP1 genes were not significantly different between the smokers and nonsmokers. However, there were significant differences (*P* = 0.05) between groups in polymorphisms in the GSTM1 gene. The patients with chronic periodontitis have a higher frequency of null and mutant polymorphisms in GSTM1, GSTT1, and GSTP1 compared with historical data from a healthy Mexican population. *Conclusions.* The presence of these polymorphisms may be a risk factor for the development of chronic periodontitis.

## 1. Introduction

According to the World Health Organization, periodontal diseases are the second most prevalent oral cavity diseases, trailing only dental caries. The latest oral health report from the World Health Organization (April 2012) indicates that between 15 and 20% of the middle-aged adult population (35–45 years) has periodontal disease [1].

Data from the Health Department of Mexico the 8.8% of Mexican population has chronic periodontitis. This is more common between subjects of 35 years and older where it is estimated that the frequency is of 22%, stressing that the age being most affected by dependence of snuff is between 35 and 65 years [2, 3].

Chronic periodontitis is defined as the inflammation and destruction of the periodontal tissue and the alveolar bone supporting the teeth. This affects normal oral functions as mastication, speech, and facial aesthetics [1]. The progression and severity of the disease depend on complex interactions between several risk factors such as microorganisms, age, race, and immunological, environmental, and genetic factors [4].

Polymorphisms are monogenic mutations in which the substitution of a nitrogenous base with a different base pair occurs. They are considered normal variants within the population and may or may not lead to variations in phenotype [5]. Several studies have reported that different polymorphic genes are associated with risk of developing chronic periodontitis [6–11].

There have been previous studies examining such genetic changes in glutathione S-transferase genes such as GSTM1, GSTT1, and GSTP1, which are involved in the detoxification of polycyclic aromatic hydrocarbons such as epoxides and hydroxylated metabolites of benzo-a-pyrene derived from tobacco [12, 13]. Some studies indicate that smoking habits and polymorphisms in GST genes are associated with different diseases, including chronic periodontitis [14–17].

However, it is unclear whether the effect of smoking on chronic periodontitis is influenced by genetic susceptibility. Therefore, the aim of this study was to determine the frequency of GSTM1, GSTT1, and GSTP1 polymorphisms in Mexican population of smokers and nonsmokers with chronic periodontitis.

## 2. Material and Methods

### 2.1. Study Population and Clinical Examination

In total, 60 Mexican subjects were recruited for the present study. All cases were patients diagnosed with chronic periodontitis, in clinics at the Faculty of Dentistry at the Autonomous University of the State of Mexico. Patients with chronic periodontitis were classified according to the definitions used in the National Survey of Addictions in Mexico such as smoking people who reported having smoked in the past year and nonsmoking individuals who reported not smoking for a period of at least one year [18]. Both groups accepted their participation in the study by signing an informed consent designed for this purpose. Demographics and smoking habits data were obtained using a self-reported questionnaire.

This study was approved by the Ethics Committee of the Center for Research and Advanced Studies in Dentistry of the Autonomous University of the State of Mexico.

### 2.2. Diagnosis of Periodontal Disease

The patients were diagnosed according to the criteria of IWC 1999 International Workshop for a Classification of Periodontal Diseases and Conditions. The clinical and physical measurements, including the evaluation of probing depth, bleeding on probing, tooth mobility, number of teeth present, accumulation of mineralized plaque, and signs of inflammation, were recorded in order to identify severity and extent of disease [19, 20].

### 2.3. DNA Extraction

A 3 mL sample of peripheral blood from each participant was drawn into a Vacutainer tube with heparin, which was kept refrigerated until processed. DNA extraction was performed using the Quick-gDNA MiniPrep Kit (Zymo Research Corporation, USA). The products were verified by horizontal electrophoresis in 1% agarose and stored at −15°C until the amplification.

### 2.4. Genotyping of GSTM1 and GSTT1 Polymorphisms

Identification of polymorphisms of GSTM1 and GSTT1 was performed by multiplex PCR with CYP1A1 gene as control. The mixture had a final volume of 25 *μ*L, containing 5 *μ*L 5x PCR buffer, 1.5 *μ*L of 25 mM MgCl_2_, 1 *μ*L 50 *μ*M dNTP, 9.4 *μ*L of molecular biology grade H_2_O, 0.1 *μ*L 5 U/*μ*L Taq Polymerase (Promega, Madison, WI, USA), 2 *μ*L of DNA template, and 1 *μ*L of primers CYP1A1-f, CYP1A1-r, GSTM1-f, GSTM1-r, GSTT1-f, and GSTT1-r, all primers had a concentration of 30 pm (Table 1) [21]. PCR conditions for these amplifications were 5 minutes at 94°C, denaturation 1 minute at 94°C, alignment 1 minute at 59°C, extension 1 minute at 72°C, and a final elongation of 5 minutes at 72°C, for 35 cycles. PCR products were verified by horizontal electrophoresis using 2% agarose.

**Figure 1:**
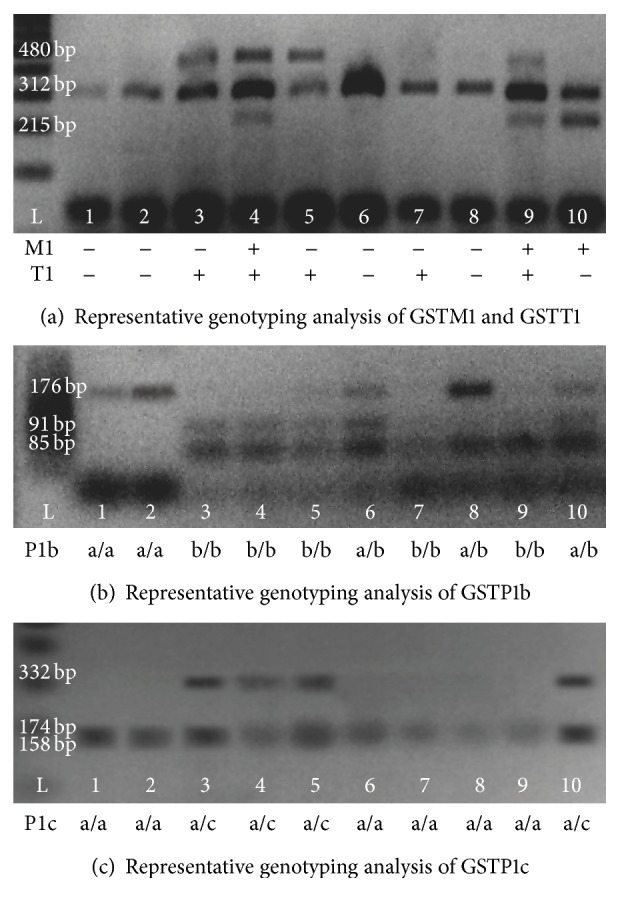
Showing an example of 10 results obtained from the amplification of the GSTM1 and GSTT1 genes using the CP1A1 gene as a control. Additionally, gels showing the results of GSTP1b and GSTP1c detection.

The determination of the polymorphism of GSTT1 was based on the presence of a 480 bp band, corresponding to wild GSTT1, while its absence denoted a null GSTT1. Similarly, for GSTM1, the presence of a 215 bp band indicated wild GSTM1, while its absence implied null GSTM1. Finally, a 312 bp band, corresponding to CYP1A1, was always present and was employed as a PCR control gene (Figure 1).

### 2.5. Genotyping of GSTP1 Polymorphisms

Identification of GSTP1b (exon 5) and GSTP1c (exon 6) was carried out in two separate PCRs, one for each polymorphism, both with a final volume of 25 *μ*L in order to obtain a sufficient amount of product for subsequent enzymatic digestions. The mixture for the PCR reaction contained 2 *μ*L of template DNA, 5 *μ*L of 5x PCR buffer, 1.5 *μ*L of 25 mM MgCl_2_, 0.1 *μ*L of 5 U/*μ*L Taq Polymerase, 0.5 *μ*L 50 *μ*M dNTPs, 13.9 *μ*L molecular biology grade H_2_O (Promega, Madison, WI, USA), and 1 *μ*L of each primer according to the gene to be amplified: GSTP1b-f, GSTP1b-r, GSTP1c-f, or GSTP1c-r; all primers had a concentration of 30 pm (Table 1) [22]. For GSTP1c PCR, the conditions were the same as those used for GSTT1 and GSTM1, while conditions for GSTP1b PCR were 5 minutes at 94°C, denaturation 1 minute at 94°C, alignment 1 minute at 62°C, extension 1 minute at 72°C, and a final elongation of 5 minutes at 72°C for a total of 35 cycles. For the identification of P1 polymorphisms, enzymatic digestions were carried out using BsmAI for P1b (exon 5) and AciI for P1c (exon 6). The digestion mixture consisted of 17 *μ*L molecular biology grade H_2_O, 2 *μ*L Fast-Digest Green buffer, 1 *μ*L Fast-Digest Enzyme (Fermentas), and 10 *μ*L PCR product DNA, for a total volume of 20 *μ*L. Both digestion reactions were incubated at 37°C for 5 minutes.

The digestion products were verified by horizontal electrophoresis, using a 2% concentration of agarose. The identification of polymorphisms was based on the presence of DNA fragments of different sizes. For exon 5, 176 bp, 91 bp, and 85 bp fragments correspond to heterozygote P1b (a/b) while 91 bp and 85 bp fragments correspond to homozygote P1b (b/b) and a 176 bp fragment corresponds to wild type P1b (a/a). Regarding exon 6 polymorphisms, the 332 bp fragment corresponds to homozygote P1c (c/c); three fragments, 332 bp, 174 bp, and 158 bp, correspond to heterozygote P1c (a/c) and two fragments, 158 bp and 174 bp, correspond to wild type P1c (a/a) (Figure 1).

### 2.6. Statistical Analysis

To differentiate between the frequencies of each polymorphism the analysis performed was different in proportions tested due to the nature of the variables. Statistical analysis was performed using SPSS software support in the 20 version (SPSS 20.0; SPSS Inc., Chicago, IL, USA).

## 3. Results

### 3.1. Study Population

The demographic and clinical characteristics of the subjects included in the study are presented in Table 2. The mean age of the nonsmokers was 53.43 ± 13.6 years, and the mean age of the smokers was 44.43 ± 7.39 years with a range of 25–72 years for all patients. With regard to the level of education, no differences were observed between the groups.

Regarding the characteristics of the disease found among smokers, there was an average loss of 1.8 more teeth compared to the nonsmokers. The results for the full mouth plaque score, calculus, and severity of the chronic periodontitis were very similar between groups.

### 3.2. Results of Polymorphisms of GSTM1, GSTT1, and GSTP1

The results of the analysis of polymorphisms in GSTM1 and GSTT1 indicated that there was a higher incidence of the GSTM1 null allele of 66.7% in the nonsmokers group compared to 43.4% with the GSTM1 null allele in the smokers group. Most individuals in both groups had the GSTT1 null polymorphism; the percentages were 73% of nonsmokers and 56.7% of smokers. The results of the identification of GSTP1 polymorphisms were very similar. It was observed that the GSTP1b a/a genotype was only present in the smokers group with an incidence of 10%. No cases of the GSTP1c polymorphism were found in the homozygous form c/c in both groups (Table 3).

When comparing the proportions of the polymorphisms between the smokers and the nonsmokers, no significant difference between the groups was observed for GSTT1 and GSTP1. No differences in the GSTM1 gene were expected; however, significant difference between the groups was observed for this polymorphism (*P* ≤ 0.05) (Table 3).

As no differences between the groups were found in the majority of the genes studied, the patients with chronic periodontitis were grouped regardless of whether they smoked or not. With such grouping, the data obtained show that for both GSTM1 and GSTT1, there is a higher incidence of null polymorphisms. The results of the combined genotype analysis show that 80% of the patients have a null allele, that is, combinations −/−, −/+, +/−, with the double null genotype being the most frequent, representing 40% (Table 4). This condition is characterized by the absence of specific enzymatic activity and subsequent reduced ability to detoxify potentially toxic substances [16].

Similarly, when analyzing the total population with chronic periodontitis, the data indicate that for polymorphism GSTP1b, only 5% of patients have the a/a genotype, while the remaining 95% have some sort of variation. In contrast, 81.6% of patients had the a/a genotype for the GSTP1c polymorphism. No patients with the genotype c/c were reported (Table 4).

## 4. Discussion

The analysis of gene-environment interactions may be the best way to understand the increased susceptibility to chronic periodontitis. Inflammation is known to be a consequence of periodontal bacteria and harmful products derived from cigarette smoke. Additionally, GST is involved in the detoxification of toxins that cause oxidative stress; some studies suggest an association between chronic periodontitis and polymorphisms in genes encoding enzymes that metabolize compounds derived from tobacco smoke [7, 16, 17].

Several authors have reported that smoking is a risk factor for periodontitis [23–27]. We compared patients who are smokers against those who are nonsmokers and found a very similar degree of disease severity between the groups. This result should be considered with caution because this study only examined the smoking habit during the last year, which gives the rise to considering some subjects who are recently quitters within the sample of nonsmokers. According to the literature, individuals with a lower education level are more likely to develop chronic periodontitis, which does not agree with this study [1, 4].

By combining the smoker and nonsmoker groups, we created a total sample of 60 Mexican subjects with chronic periodontitis. In this sample, 55% of patients had the GSTM1 null genotype, which is consistent with the data reported by Concolino et al. (2007). These authors found an increased presence of the GSTM1 null genotype in 73.9% of their study group comprising of Caucasians from central Italy with chronic periodontal disease, similar to our data. Both of these studies differ from studies in a Korean population conducted by Kim et al. (2004), who reported this allele in just 37% of their subjects. For the GSTT1 null genotype, our results showed a frequency of this allele in 65% in our patients, which differs from the Caucasian population data being 24.6% for this allele [16, 17].

Montero et al. (2007) performed a research study to determine the frequencies of polymorphisms in GSTM1 and GSTT1 in a healthy Mexican population with parents and grandparents born in Mexico. These authors had a study population comprising of 150 individuals, and they found that GSTM1 null is present with a frequency of 45% and that GSTT1 null has a frequency of 9.7%. Pérez-Morales et al. (2008) performed a similar study comprising of 529 Mexicans, and these authors observed a frequency of 33.4% for GSTM1 null and 12% for GSTT1 null [28, 29].

Comparing these data and our results using a population of 60 Mexicans with chronic periodontitis, an increase in the frequency of the GSTM1 null allele to 55% is observed and our data indicate that the GSTT1 null allele is present at a frequency of 65%. Moreover, there is also an increase in the frequency of the combined GSTM1/T1 double null genotype, which was present in 40% of our patients compared to 2.2% and 4.24% reported by Pérez and Montero, respectively. This is consistent with the hypothesis that the double null combination leads to a decreased capacity to detoxify, which could lead to an increased susceptibility to chronic periodontal disease.

There are no data in any population showing the prevalence of GSTP1b and GSTP1c in relation to chronic periodontitis, but there are data on these genes in healthy populations. A study performed by Mejia et al. (2013) in 60 Mexican subjects reported frequencies for GSTP1b and GSTP1c polymorphisms. They reported that for the frequencies of the wild type P1b allele a/a was 33.3%; the frequency of the heterozygous P1b allele a/b was in 56.6%, and the frequency of the homozygous P1b allele b/b was 10% [22].

Comparing both studies, we observed a decrease in the presence of the wild type allele a/a and an increase in the homozygous allele b/b in our group of patients. Additionally, it is notable that 95% of the sick population of patients had some polymorphism in this gene. With regard to the distribution of GSTP1c, Mejia et al. reported that 100% of the individuals in their study had the wild type genotype a/a [22], while in our study we found that 81.6% of patients were wild type a/a and 18.4% of patients were heterozygous a/c. This shows a decreased capacity to detoxify in patients with chronic periodontitis compared with historical data from a healthy population.

The observed frequencies of GSTM1 and GSTT1 nulls, GSTP1b and GSTP1c mutants show that these genes may confer increased susceptibility to chronic periodontal disease.

A better understanding of the function of the polymorphic genes GSTM1, GSTT1, and GSTP1 and a better understanding of the disease could contribute to the prevention of chronic periodontitis through personalized recommendations and specific intervention.

In conclusion, within the limitations of this study, the results suggest that compared with historical data from a healthy Mexican population, the population with chronic periodontitis shows an increase in null and mutant polymorphisms in GSTM1, GSTT1, and GSTP1. Therefore, the presence of these polymorphisms may be a risk factor for the presence of chronic periodontitis.

Importantly, the results of this pilot study comprise the first report of the presence of GSTM1, GSTT1, and GSTP1 polymorphisms in a Mexican population with chronic periodontitis. The future development of this research will be the realization of these tests in a larger group of patients to measure the relationship between susceptibility genes and chronic periodontitis.

## Figures and Tables

**Table 1:** Sequences of primers used for end-point PCR.

Genes	Sequence	Amplicon
GSTM1	F-5′-GTTGGGCTCAAATATACGGTGG-3′	215 bp
	R-5′-GAACTCCCTGAAAAGCTAAAGC-3′	

GSTT1	F-5′-TCACCGGATCATGGCCAGCA-3′	480 bp
	R-5′-TTCCTTACTGGTCCTCACATCTC-3′	

CYP1A1	F-5′-GAACTGCCACTTCAGCTGTCT-3′	312 bp
	R-5′-CAGCTGCATTTGGAAGTGCTC-3′	

GSTP1b	F-5′-ACCCCAGGGCTCTATGGGAA-3′	176 bp
	R-5′-TGAGGGCACAAGAAGCCCCT-3′	

GSTP1c	F-5′-TGGCAGCTGAAGTGGACAGGATT-3′	332 bp
	R-5′-ATGGCTCACACCTGTGTCCATCT-3′	

**Table 2:** Sociodemographic and disease characteristics of the study groups.

	Nonsmokers *n* = 30	Smokers *n* = 30
Age (years)		
Mean ± SD	53.43 ± 13.6	44.43 ± 7.39
(interval)	(25–72)	(28–62)
Gender (%)		
Male	16 (53.3)	26 (86.7)
Female	14 (46.7)	4 (13.3)
Education (%)		
Illiterate	1 (3.3)	2 (6.7)
Elementary School	12 (40)	10 (33.3)
High school	9 (30)	5 (16.7)
College	4 (13.3)	6 (20)
Professional	4 (13.3)	7 (23)
^ a^FMPS		
Mean ± SD	54.20 ± 17.68	48.0 ± 14.21
(interval)	(28–97)	(26–80)
Calculus		
Mean ± SD	22.80 ± 13.42	20.67 ± 7.19
(interval)	(4–68)	(8–34)
Teeth present		
Mean ± SD	25.63 ± 4.38	23.83 ± 4.19
(interval)	(13–31)	(12–30)
Disease severity (%)		
Mild	11 (36.7)	12 (40)
Moderate	14 (46.7)	14 (46.7)
Serious	5 (16.7)	4 (13.3)

^a^Full mouth plaque score.

**Table 3:** Results of polymorphisms in GST M1, T1, P1b, and P1c of the study groups.

Genotype	Nonsmokers *n* = 30 (%)	Smokers *n* = 30 (%)
M1		
+	^ b^10 (33.3)	17 (56.7)
−	^ b^20 (66.7)	13 (43.4)
T1		
+	8 (26.7)	13 (43.4)
−	22 (73.3)	17 (56.7)
P1b		
a/a	0 (0)	3 (10)
a/b	18 (60)	15 (50)
b/b	12 (40)	12 (40)
P1c		
a/a	24 (80)	25 (83.3)
a/c	6 (20)	5 (16.7)
c/c	0 (0)	0 (0)

^b^ *P* ≤ 0.05.

**Table 4:** Results of polymorphisms in GST M1, T1, P1b, and P1c of patients with chronic periodontitis.

Genotype	*n* = 60 (%)
M1	
+	27 (45)
−	33 (55)
T1	
+	21 (35)
−	39 (65)
M1 + T1	
−/−	24 (40)
−/+	9 (15)
+/−	15 (25)
+/+	12 (20)
P1b	
a/a	3 (5)
a/b	33 (55)
b/b	24 (40)
P1c	
a/a	49 (81.6)
a/c	11 (18.4)
c/c	0 (0)
